# In vitro reconstitution of SPO11‐mediated DNA cleavage reveals mechanistic and clinical insights into meiotic DNA double‐strand break formation

**DOI:** 10.1002/ctm2.70383

**Published:** 2025-06-22

**Authors:** Zetao Hu, Xinzhe Tang, Ming‐Han Tong, Ying Huang

**Affiliations:** ^1^ Department of General Surgery Shanghai Key Laboratory of Biliary Tract Disease Research Xinhua Hospital Shanghai Jiao Tong University School of Medicine Shanghai China; ^2^ Key Laboratory of Multi‐Cell Systems Shanghai Key Laboratory of Molecular Andrology Shanghai Institute of Biochemistry and Cell Biology Center for Excellence in Molecular Cell Science Chinese Academy of Sciences University of Chinese Academy of Sciences Shanghai China

**Keywords:** germ cell, infertility, meiosis, SPO11

1

Infertility affects 8–12% of couples worldwide, with male factors contributing to up to 50% of cases. Notably, approximately 30% of couples are classified with unexplained infertility despite having normal karyotyping, hormone levels, and semen parameters.^1^ One overlooked but fundamental cause is defective meiosis—‐the specialized cell division that halves the chromosome number to produce haploid gametes.^2^, ^3^ A critical step in meiosis is the formation of programmed DNA double‐strand breaks (DSBs), catalyzed by the topoisomerase‐like nuclease SPO11, which licenses homologous recombination and chromosome synapsis.^4^ SPO11 is essential for meiosis: in mice, for instance, *Spo11*‐*null* males are arrested before the pachytene stage, and females lose nearly all oocytes shortly after birth.^5^ However, the catalytic architecture of human SPO11, its tolerance to activity loss, and the clinical significance of rare alleles remain poorly defined.

To address this, we established an in vitro reconstitution system for mouse SPO11‐mediated DSB formation, confirming its catalytic activity outside a cellular context.^6^ Notably, two other groups independently reported similar findings in parallel studies.^7^, ^8^ Biochemical characterization revealed that although the purified SPO11 and its partner TOP6BL predominantly exist as heterodimers, cross‐linking mass spectrometry, pull‐down assays, and co‐immunoprecipitation (Co‐IP) identified a minor population of heterotetrameric complexes.^6^ This tetrameric assembly is widely recognized as the core catalytic framework required for DNA cleavage.

Incubation of the purified SPO11‐TOP6BL complex with both supercoiled and linear DNA substrates resulted in detectable DSBs via gel electrophoresis. Treatment of the reaction products with proteinase K or human TDP2, a tyrosyl‐DNA phosphodiesterase, demonstrated that SPO11 forms a covalent phosphotyrosyl linkage with the 5′ end of the cleaved DNA, consistent with its topoisomerase‐like activity. Structural alignment between the AlphaFold 3 predicted SPO11 complex and African swine fever virus topoisomerase II (AsfvTop2) revealed two adjacent conserved residues, Y137 and Y138.^9^ Site‐directed mutagenesis demonstrated that only Y138—‐not Y137—‐is essential for forming the covalent linkage with DNA, identifying Y138 as the catalytic nucleophile. This was further supported by biochemical assays using 46 bp Cy3‐labeled DNA substrates. These findings provide a direct molecular visualization of SPO11's catalytic mechanism and significantly advance the understanding of meiotic recombination.

Further structural alignment highlighted three conserved acidic residues (E224, D277 and D279) likely involved in Mg^2+^ coordination essential for catalysis. Mutational analyses revealed that D277N substitution nearly abolished activity, while E224Q and D279N retained partial function. To evaluate the physiological impact, we generated *Spo11^D277N/D277N^* knock‐in mice via CRISPR/Cas9. At 19 days postpartum (dpp), mutant testes exhibited meiotic arrest at a zygotene‐like stage with increased apoptosis, whereas heterozygous controls showed normal spermatogenesis. In mutant females, primordial follicles were nearly absent by 12dpp. Immunostaining displayed a loss of DSB markers (RPA2, γH2AX) and defective synapsis (SYCP1), phenocopying *Spo11^⁻/⁻^* mouse phenotypes. These findings establish that SPO11's catalytic activity—and thus DSB formation—is indispensable for early meiotic progression and germ cell development (Figure 1).

**FIGURE 1:**
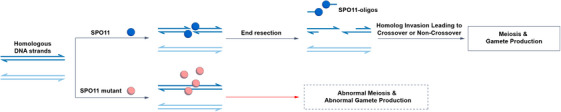
SPO11‐mediated homologous recombination pathway during meiosis and consequences of SPO11 mutation. SPO11 introduces DSBs on homologous DNA strands, initiating recombination. After DSB formation, end resection generates single‐stranded DNA overhangs, releasing SPO11–oligonucleotide complexes. These overhangs facilitate homolog invasion, resulting in either crossover or non‐crossover recombination, and proper meiosis with gamete production. In contrast, SPO11 mutants fail to introduce DSBs effectively, preventing proper recombination and leading to abnormal meiosis and defective gamete formation.

In parallel, human genetic studies have linked SPO11 variants to infertility, underscoring the gene's clinical relevance. A meta‐analysis of five case‐control studies (542 infertile men, 510 controls) found that the C631T polymorphism (rs28368082) is associated with increased male infertility risk across multiple genetic models.^10^ The odds ratios were consistently elevated: 4.14 under the dominant model (TT+CT vs. CC), 4.34 in the heterozygous model (CT vs. CC), and 4.35 for the allelic model (T vs. C), with confidence intervals ranging from 2.48 to 7.34. Subgroup analysis demonstrated that this association was statistically significant in Chinese populations (*p* < .01) but not in Iranian cohorts (*p* > .05), suggesting population‐specific genetic susceptibility.^10^

A mouse model carrying the human‐equivalent *P306T* missense mutation in *spo11* (*Spo11^P306T/P306T^*) exhibited oligospermia in males and diminished ovarian reserve in females, due to delayed DSB formation.^5^ Mutant mice showed synapsis abnormalities (particularly X‐Y pairing failure), elevated apoptosis, persistent DSB markers, and reduced Type I crossovers, leading to achiasmate chromosomes. These defects were further exacerbated in *Spo11^P306T/‐^* mice, which were sterile with severely impaired DSB formation. These results confirm that *P306T* is a hypomorphic allele that delays DSB initiation. In humans, this variant could manifest as premature ovarian insufficiency, oligozoospermia, and elevated aneuploidy risk, underscoring the necessity of functional validation of variants of uncertain significance (VUS) in reproductive genes.^5^

In summary, the initiation of meiotic recombination through SPO11‐mediated DSB formation is essential for gametogenesis. Our study demonstrates that SPO11 forms dimers in vitro and covalently binds to DNA at the 5′ ends of its cleavage products. Furthermore, its catalytic activity is indispensable for meiosis and germ cell development (Figure 1). Disruption of this activity—whether by targeted mutagenesis or naturally occurring human genetic variants—leads to meiotic arrest and gametogenic failure. This work establishes an in vitro platform to functionally validate DSB‐related gene variants implicated in human infertility and to elucidate the biochemical mechanisms underlying the regulation of DSB formation in vivo.

## CONFLICT OF INTEREST STATEMENT

The authors declare no conflict of interest.

## ETHICS STATEMENT

Not applicable.

## Data Availability

Data sharing is not applicable to this article as no new data were created or analyzed in this study.
